# Les déficits visuels chez les conducteurs de taxi dans la ville de Niamey

**DOI:** 10.11604/pamj.2020.37.273.25014

**Published:** 2020-11-25

**Authors:** Hadjia Yakoura Abba Kaka, Roufaye Lamyne, Nephthali Gnangourou, Amza Abdou

**Affiliations:** 1 Service d´Ophtalmologie, Hôpital National de Niamey, Niamey, Niger,; 2 Service d´Ophtalmologie, Hôpital National Lamordé Niamey, Niamey, Niger

**Keywords:** Conduite automobile, aptitude visuelle, déficits visuels, taxi, Niamey

## Aux éditeurs du Pan African Medical Journal

La conduite automobile est réglementée selon la législation qui prévaut dans chaque pays. On peut distinguer des déficits visuels rendant le conducteur inapte à la conduite et ceux compatibles à la conduite automobile [1,2]. La sécurité routière, celle des usagers et celles des conducteurs passe par une aptitude visuelle optimale. L´évaluation de celle-ci est donc primordiale avant l´octroi du permis de conduire toute catégorie confondue. C´est dans cette optique que les auteurs dans une étude transversale et descriptive du 31 octobre 2016 au 31 janvier 2017 se sont fixé comme objectif d´évaluer la fréquence des déficits visuels chez les conducteurs de taxi dans la capitale Niamey en tenant compte de l´acuité visuelle et du champ visuel. Nous avons inclus les conducteurs de taxi de la ville de Niamey désireux de participer à l´étude à travers le syndicat des conducteurs de taxi, étaient non inclus les anciens chauffeurs de taxis et ceux non consentants à l´étude.

La méthode probabiliste a été utilisée par la technique de sondage aléatoire simple afin de choisir les conducteurs. La taille de notre échantillon était limitée à 200 conducteurs. Des cartes de consultation ont été distribuées à 200 conducteurs de taxi pris au hasard, qui ont été enregistrés sur une liste avec chacun un numéro d´anonymat et son numéro de téléphone. Ils ont été ensuite convoqués par groupe de cinq pour un examen ophtalmologique. L´interrogatoire a précisé: l´identité, la catégorie de permis de conduire, l´examen oculaire avant obtention du permis ou au cours de la profession, la correction optique, l´acuité visuelle de loin monoculaire et binoculaire a été mesurée avec l´optotype Monoyer ou Snellen à 5m. L´examen du segment antérieur a été fait à la lampe à fente avec mesure de la pression intraoculaire au tonomètre de Goldman.

Tous nos patients ont bénéficié d´un champ visuel à la coupole de Goldmann. Une fiche d´enquête nous a servi de support de collecte de données. Les données ont été saisies, traitées et analysées à l´aide des logiciels Microsoft office 2010 (Word et Excel), Epi Info version 3.5.4. Au total 62 conducteurs tous de sexe masculin ont répondu aux critères d´inclusion. L’âge des patients variait de 24 à 63 ans, la tranche d´âge 40-49 ans a représenté 53,2%. La moyenne d´âge était de 45,62 ans. Tous nos patients avaient un permis de conduire catégorie B et 55 cas soit 89% n´ont pas subi un examen oculaire avant la délivrance de leur permis de conduire. Un peu plus de neuf pourcent des conducteurs avaient une acuité visuelle binoculaire inférieure à 5/10^e^, 21% des cas avaient un champ visuel binoculaire horizontal inférieur à 120 degré. Treize conducteurs soit 21% avaient un champ visuel binoculaire horizontal inférieur à 60°. Un déficit visuel a été retrouvé chez 15 conducteurs soit 24% et 21% de ceux-ci avaient un déficit visuel les rendant inaptes à la conduite de taxi (soit 1 conducteur sur 5).

La cataracte a représenté 46,15% des causes de déficits visuels suivie des amétropies non corrigées dans 26%. Les conducteurs aptes à la conduite étaient de la tranche d´âge 40-49 ans avec 45,14%. Ceux ayant des déficits visuels les rendant inaptes étaient plus représentés dans la tranche d´âge 50-59 ans avec 9,69%. Une bonne faculté visuelle est indispensable pour une conduite sécuritaire. Toute atteinte importante de la fonction visuelle, de l´acuité visuelle ou du champ visuel, pour les plus connues, diminue l´aptitude d´une personne à conduire sur les routes d´aujourd´hui [3]. Nous n´avons pas retrouvé dans la littérature de lien entre le nombre d´accidents de circulation secondaire à des défauts optiques non corrigés ou à des pathologies visuelles. La législation du Niger, datant de 1963 est en projet de réactualisation par les autorités en charge du transport publique et doit plus fermement tenir compte de l´aptitude visuelle avant l´octroi de tout permis de conduire toute catégorie confondues [4-6].

De cette présente étude il en ressort qu´une grande partie des conducteurs de taxi n´avaient jamais subi un examen oculaire avant la délivrance et/ou le renouvellement de leur permis de conduire et plusieurs avaient un déficit visuel les rendant inaptes à la conduite. Au vu de ces résultats, il serait souhaitable de mener une étude à grande échelle au Niger pour détecter les déficits visuels chez les conducteurs et éventuellement les corriger. De façon unanime toutes les études ont pour recommandation la mise en place d´un système imposant l´évaluation de l´aptitude visuelle avant l´octroi du permis de conduire et l´optimisation périodique de la correction visuelle des amétropes. La sécurité des routiers et celle des autres dépend strictement de la vision de celui au volant. Véritable problème de santé publique les accidents de la voie publique font des victimes par des causes évitables.

## Remerciements

Au SYNCOTAXI (syndicat des conducteurs de taxi) de la ville de Niamey et au Ministère de Transport du Niger.
